# *Siniperca chuatsi* Rhabdovirus (SCRV)-Induced Key Pathways and Major Antiviral Genes in Fish Cells

**DOI:** 10.3390/microorganisms10122464

**Published:** 2022-12-13

**Authors:** Fei Ke, Xian-Yu Meng, Qi-Ya Zhang

**Affiliations:** 1 Institute of Hydrobiology, College of Modern Agriculture Sciences, University of Chinese Academy of Sciences, Chinese Academy of Sciences, Wuhan 430072, China; 2 The Innovation Academy of Seed Design, Chinese Academy of Sciences, Beijing 100101, China

**Keywords:** *S. chuatsi* skin cell line (SCSC), *S. chuatsi* rhabdovirus (SCRV), transcriptome response, cytokines, interferon-stimulated genes

## Abstract

Fish rhabdoviruses, including *Siniperca chuatsi* rhabdovirus (SCRV), are epidemic pathogens that harm fish aquaculture. To clarify the interactions between SCRV and its host and explore antiviral targets, the present study performed transcriptome analysis in a cultured *S. chuatsi* skin cell line (SCSC) after SCRV infection at 3, 12, 24, and 36 h post-infection (hpi). Comparison with control obtained 38, 353, 896, and 1452 differentially expressed genes (DEGs) in the detected time points, respectively. Further analysis of the Go terms and KEGG pathways revealed the key pathways “Cytokine-cytokine receptor interaction” and “interferon related pathways” in SCSC cells responding to SCRV infection. The significantly up-regulated genes in the pathways were also verified by qPCR. Furthermore, gene cloning and overexpression revealed that five interferon-stimulated genes (ISGs) IFI44_07_, IFI35, Viperin, IFIT1, and IFIT5 had the ability to inhibit SCRV replication in FHM (Fathead minnow) cells, especially an inhibition efficiency more than 50% was observed in IFI35 overexpressed cells. In summary, current study revealed the main innate immune pathways in *S. chuatsi* cells induced by SCRV infection and the major ISGs of *S. chuatsi* in controlling SCRV replication.

## 1. Introduction

Aquaculture has become one of the fastest-growing agricultural and food production industries in the world [1]. The culture of fish is an important constituent part of aquaculture [2]. *Siniperca chuatsi* (also known as Mandarin fish or Chinese perch) is an economically important fish cultured in China with high market value, and it belongs to the family *Serranidae* in the order Perciformes [3,4]. However, the *S. chuatsi* aquaculture industry has been hampered by diseases caused by viruses such as rhabdoviruses [5].

The family *Rhabdoviridae* contains viruses with a bullet-shaped virion and negative-sense single-stranded RNA genome [6]. *S. chuatsi* rhabdovirus (SCRV) was first isolated from diseased mandarin fish in 1999 in China [7], which has a genome of 11545 nucleotides encoding five structural proteins [8]. Isolates of SCRV have been reported in recent years for the outbreak of related diseases [9], which was even found to coinfect with other viruses in mandarin fish [10]. Knowledge of host responses to virus infection will benefit antiviral research. High-throughput sequencing technologies have been used in the exploration of the the host responses of virus-infected *S. chuatsi*. For example, Huang et al. investigated the transcriptome responses of *S. chuatsi* at 24 h (h) and 60 h post-infection (hpi) with SCRV [11], which revealed several pathways involved in SCRV infection in vivo. In addition, deep sequencing revealed the changes in microRNAs profiles in SCRV-infected Chinese perch brain cells [12]. However, the transcriptome responses of a cultured *S. chuatsi* cell line during SCRV infection are still lacking. More research on the genes of *S. chuatsi* that function against SCRV infection is still needed.

Recently, we established a *S. chuatsi* skin cell line (SCSC), which showed high sensitivity to SCRV infection [13]. In the present study, cellular responses of SCSC to SCRV infection were investigated by transcriptome analysis, which revealed several important pathways and genes. The antiviral ability of several genes was then evaluated, which revealed the major anti-SCRV interferon-stimulated genes (ISGs).

## 2. Materials and Methods

### 2.1. Cells and Virus

The SCSC cell line and the FHM (Fathead minnow) cell line have been preserved in our laboratory [13,14]. SCRV was isolated by our lab from diseased *S. chuatsi* [8]. In the present study, SCSC cells were cultured in L15 medium supplemented with 10% FBS (fetal bovine serum) at 25 °C. FHM cells were cultured in M199 medium supplemented with 10% FBS at 25 °C. The FHM cells have higher transfection efficiencies than SCSC and were also sensitive to SCRV infection, so they were used in the transfection assays in the present study.

### 2.2. Virus Infection

For preparing virus stocks, SCSC cells were infected by SCRV at a MOI (multiplicity of infection) of 0.1. The cells were harvested when about 80% of cells had a cytopathic effect (CPE). After freezing and thawing three times, they were divided into small pieces and incubated at −80 °C until use. For preparing the infected cells used in RNA-seq, SCSC cells were infected by SCRV prepared above at a MOI of 1, and then were collected at 0, 3, 12, 24, and 36 h post-infection (hpi) and subjected to RNA extraction.

### 2.3. RNA Isolation and Sequencing

Total RNA of the collected cells (group SCRV-0 h, SCRV-3 h, SCRV-12 h, SCRV-24 h, and SCRV-36 h, *n* = 3) was extracted by using TRIzol reagent (Invitrogen, Carlsbad, CA, USA). RNA quality, including purity, integrity, and concentration, was examined by using a NanoDrop 2000 spectrophotometer (ThermoFisher, Waltham, MA, USA) and electrophoresis. The TruseqTM RNA sample prep kit (Illumina, San Diego, USA) was used to construct libraries with the RNA samples with high quality. In brief, mRNA was enriched with poly-T magnetic beads and then fragmented. cDNA was synthesized, and their cohesive ends were repaired. Adapters were ligated to the 3′ ends after the addition of adenosines. PCR was used to enrich the cDNA fragments, and the products were purified by electrophoresis and quantified using Qubit 2.0 and Agilent 2100 bioanalyzer. Then, the libraries were sequenced on MGI DNBseq-T7 in 150 bp paired-end mode, and raw data reads were generated.

### 2.4. Data Analysis

Poor-quality reads, adapters, and poly-N were deleted from the raw reads by using software Trimmomatic to obtain clean reads. Data quality was evaluated by calculating the Q20, Q30, and GC contents. Aligning the clean reads to the reference genome of *S. chuatsi* (ASM2008510v1) was performed with HISAT2 (v2.1.0) software. Expression analysis was performed with RSEM software, and the number of reads mapped to each transcript of each sample was obtained, then, FPKM (Fragments Per Kilobase Per Million bases) conversion was performed.

### 2.5. Differential Expression Analysis

DESeq2 package was used to perform the differential expression analysis based on aligned transcripts (unigenes) obtained above and expression levels obtained from RSEM software. The unigenes with |log2Fold Change| (|log2FC|) > 1 and FDR < 0.05 were assigned as differentially expressed genes (DEGs). Gene ontology (Go) annotation and pathway analysis were performed using blast2go software and Kyoto Encyclopedia of Genes and Genomes (KEGG) database, respectively. Goatools and KOBAS were used to perform enrichment analyses of Go terms and KEGG pathways based on Fisher’s exact test, respectively. *p*-values were corrected by using multiple corrections, including Bonferroni, false discovery rate, Holm, and Sidak. DEGs with a corrected *p*-value ≤ 0.05 were considered significantly enriched.

### 2.6. Experimental Detection of DEGs by RT-qPCR

The genes involved in chemokine or interferon (IFN) signaling pathways were selected for real-time quantitative PCR (RT-qPCR) analysis. The samples used for RNA extraction were collected as described above. The HiScript III RT SuperMix for qPCR (+gDNA wiper) (Vazyme, Nanjing, China) was used to perform first-strand cDNA synthesis. RT-qPCR was performed in a Quantagene q225MX Real-Time PCR Instrument (Kubo, Beijing, China). Each qPCR mixture contained 1 μL of cDNA, 5 μL of SYBR Premix (2×), 1 μL of primers (0.5 μL for each primer), and 3 μL of ultrapure water. The qPCR conditions were conducted as described previously [15]. The β-actin gene of *S. chuatsi* was used as an internal control. The relative expression ratios of the genes were calculated by the 2^−ΔΔCT^ method. The genes and primers used in RT-qPCR were collected in Supplementary Table S1.

### 2.7. Gene Cloning and Plasmid Construction

For cloning of the genes encoding ISGs, cDNA was synthesized by using HiScript III 1st Strand cDNA Synthesis Kit (+gDNA wiper) (Vazyme, Nanjing, China) using the RNA described above. The coding sequences of the ISGs (IFI44_07_, IFI44_08_, IFI35, Viperin, IFIT1, and IFIT5) were amplified with Pfu DNA polymerase (TransGen, Beijing, China) using the cDNAs as a template. We noted that there were two genes (LOC122878107 and LOC122878108) both encoding for IFI44, and we named the two ISGs IFI44_07_ and IFI44_08_ based on the last numbers of the two genes. PCR products were ligated into pEASY-Blunt cloning vector (TransGen, China) and sequenced. The verified products were then amplified and ligated into pcDNA3.1-2 × HA vector by infusion cloning. The vector gives a 2 × HA tag on the N-terminal of the cloned ISGs, which makes it easy for expression detection by Western blot. The finally obtained plasmids were sequenced to ensure they were correct.

### 2.8. Transfection and Western Blotting

Before the transfection, FHM cells were seeded into 24-well plates. Then, the plasmids were transfected into the cells with lipofectamine 3000 according to manufacturer’s recommendations. Twenty-four hours after transfection, the cells were collected and subjected to Western blot analysis.

Western blotting was performed as described previously [16]. The samples were analyzed by a 5–20% SDS-PAGE and then transferred to a PVDF membrane (Millipore, Boston, MA, USA). A commercial anti-HA antibody (CST, Danvers, MA, USA) was used as the primary antibody, and the horseradish peroxidase (HRP)-conjugated goat anti-rabbit IgG (Abclonal, Wuhan, China) was used as the secondary antibody. The signals were detected by chemiluminescence.

### 2.9. Virus Infection and RT-qPCR Detection

FHM cells were transfected as described above. Twenty-four hours after transfection, the cells were infected by SCRV at a MOI of 0.1. The samples were collected at 24 hpi and then subjected to RNA extraction as described above. First-strand cDNA synthesis and the RT-qPCR were performed as above mentioned.

### 2.10. Statistical Analysis

The data were analyzed using GraphPad Prism 9 software with Student’s *t*-test. Significant differences were marked with * which indicated *p* < 0.05 or ** which indicated *p* < 0.01.

## 3. Results

### 3.1. SCRV Infection in SCSC Cells

**Figure 1:**
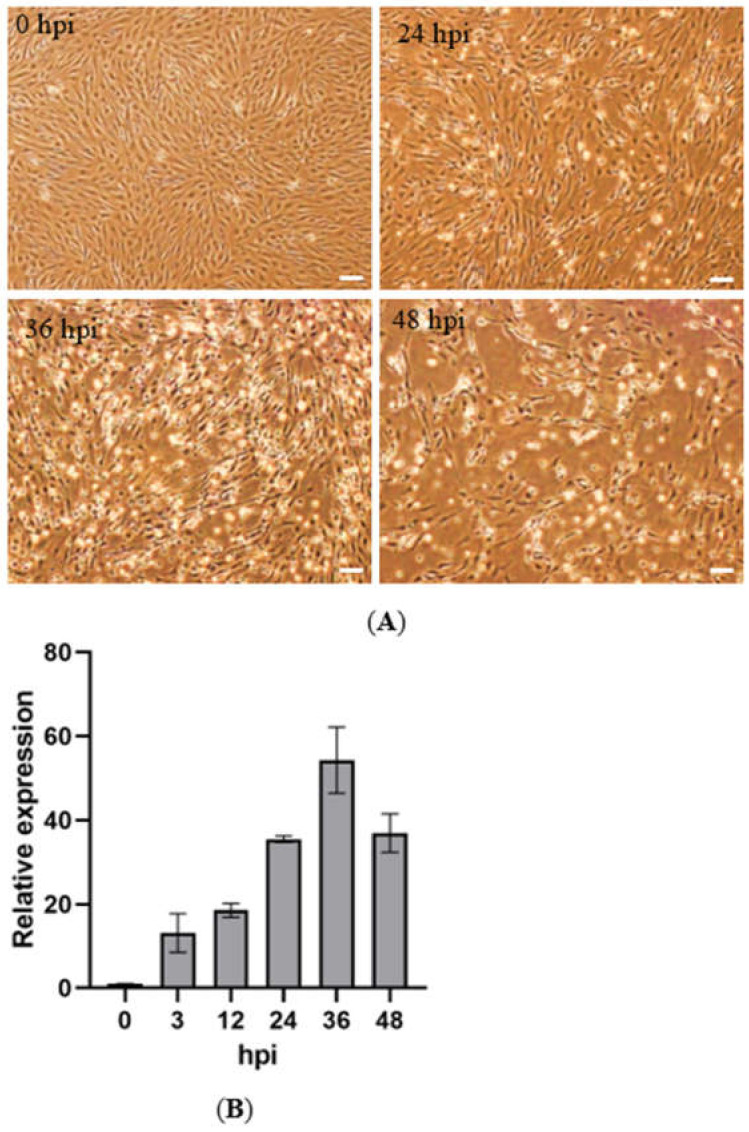
SCRV infection of SCSC cells. (**A**) Microscopy observation of the SCRV infected SCSC cells at different time points. Bar = 100 μm. (**B**) Relative expression in SCRV infected SCSC cells examined by RT-qPCR.

The cultured SCSC cells were infected with SCRV the day after their passage. As shown in Figure 1A, the SCRV-infected SCSC cells displayed CPE from 24 hpi, including the shedding and lysis of cells, which became more and more serious with the increase in the infection time. At 48 hpi, most of the adherent cells lysed or fell out. We then examined the SCRV *N* gene expression in the cells. It was shown that SCRV gene expression increased from 3 hpi to 36 hpi, while the expression level at 48 hpi was lower than that at 36 hpi (Figure 1B), possibly because of the large number of lysed cells.

### 3.2. Transcriptomic Sequence Assembly and Annotation

To further investigate the response of the SCSC cells under SCRV infection, we performed transcriptome analysis with SCRV-infected SCSC cells. The time points 0, 3, 12, 24, and 36 hpi were selected to reveal the responses. A total of 15 libraries were constructed and sequenced, which generated 151.23 Gb of clean data with more than 8.06 Gb for each sample (Table 1). The Q30 of the samples was more than 94.00%. The clean reads were then mapped to the complete genome sequence of *S. chuatsi* in the GenBank (ASM2008510v1). For most of the samples, more than 91% of the reads could be mapped to the genome, except S12-3, which had a map rate of 84.57%. In addition, more than 96% of the mapped reads were located on the exons region of the genome. The mapped results showed that a total of 26,250 genes was expressed in SCSC cells under SCRV infection, with about 20,000 genes expressed in each sample. Subsequent analysis was based on the expressed genes.

### 3.3. DEGs and Host Responses in SCRV-Infected SCSC Cells

**Figure 2:**
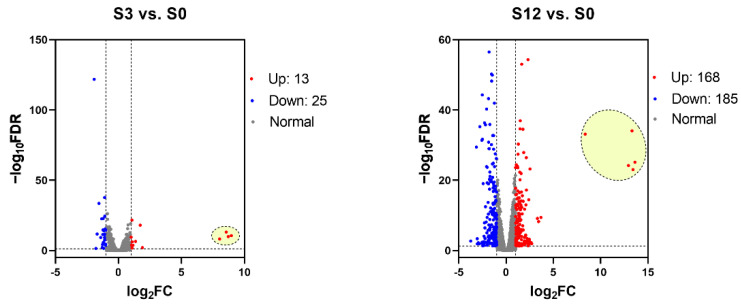

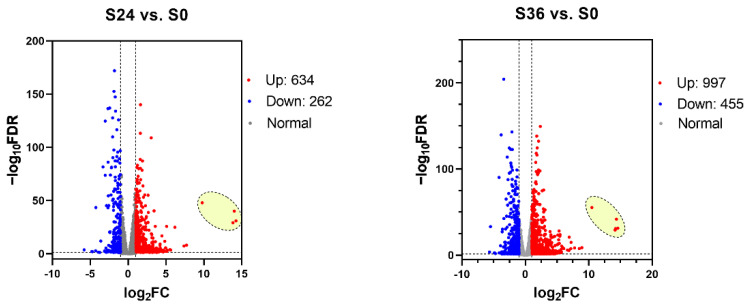
Volcano plots of DEGs in SCRV-infected SCSC cells at different time points. Up- and down-regulated DEGs are indicated by red or blue, respectively, and gray color indicates unchanged DEGs. The red plots that were boxed by yellow ellipse indicate SCRV genes.

The DEGs at different time points (3, 12, 24, 36 hpi) were analyzed by comparison with the samples at 0 hpi. The results obtained 38 (13 up-regulated), 353 (168 up-regulated), 896 (634 up-regulated), and 1452 (997 up-regulated) DEGs in the 3 (S3 vs. S0), 12 (S12 vs. S0), 24 (S24 vs. S0), and 36 (S36 vs. S0) hpi groups (Figure 2).

**Figure 3:**
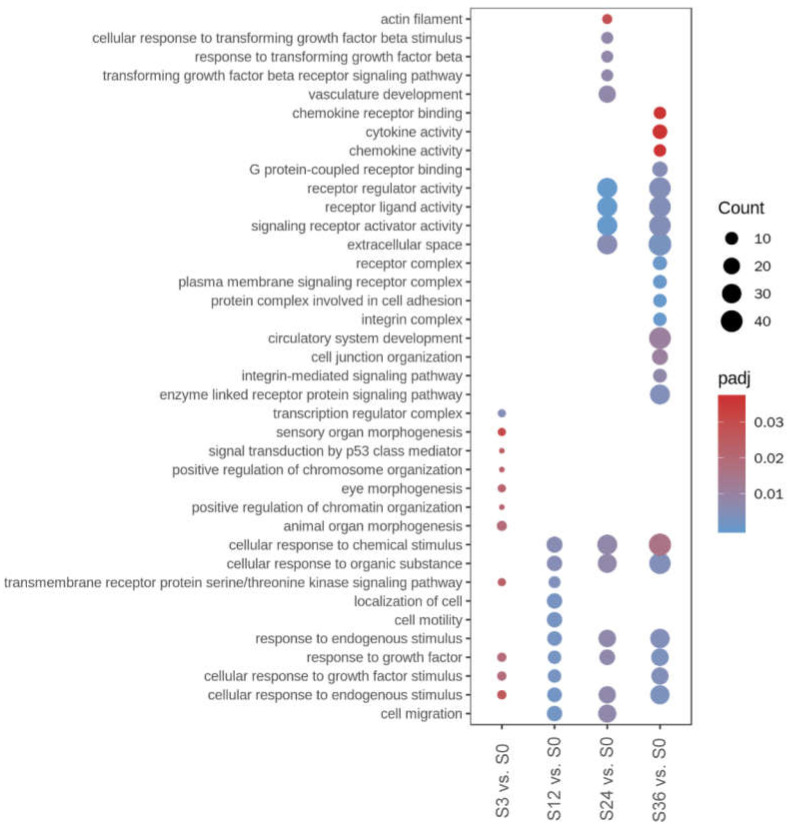
Go function enrichment analysis of DEGs. The top 10 significantly enriched Go terms from each category (BP, CC, and MF) are shown. The size of the dots indicates the number of DEGs enriched in the term (Count). The color of the dots indicates the adjusted *p*-value (padj), with a bluer color indicating smaller padj value. The terms related to “response to stimulus or growth factor” are enriched from 12 to 36 hpi. Detailed information on the enriched Go terms is collected in Tables S2–S5.

Go and KEGG pathway enrichment were further performed to analyze host responses based on the DEGs. There were 25, 20, 30, and 32 significantly enriched Go terms in the groups of S3 vs. S0, S12 vs. S0, S24 vs. S0, and S36 vs. S0, respectively (Tables S2–S5). The top 10 enriched Go terms from each category (BP, biological process; CC, cellular component; MF, molecular function) of the four groups are shown in Figure 3. The highly significantly enriched Go terms in group of S3 vs. S0 included “animal organ morphogenesis”, “cellular response to growth factor stimulus”, “response to growth factor”, and “positive regulation of chromatin organization” in the category of BP and “transcription regulator complex” in the category of CC. It was interesting that most of the enriched genes in the significantly enriched Go terms in the group of S3 vs. S0 were down-regulated. In the group of S12 vs. S0, the highly significantly enriched Go terms included “cell migration”, “cellular response to endogenous stimulus”, “cellular response to growth factor stimulus”, “response to growth factor”, and “response to endogenous stimulus” in the category of BP, which were also significantly enriched in group of S24 vs. S0. In addition, the Go terms “extracellular space”, “actin filament”, and “actin cytoskeleton” in the category of CC and “signaling receptor activator activity”, “receptor ligand activity”, and “receptor regulator activity” in the category of MF were also enriched in the group of S24 vs. S0. In the group of S36 vs. S0, the highly significantly enriched Go terms included “response to growth factor”, “cellular response to endogenous stimulus”, “cellular response to growth factor stimulus”, “response to endogenous stimulus”, and “enzyme linked receptor protein signaling pathway” in the category of BP, “integrin complex”, “protein complex involved in cell adhesion”, “plasma membrane signaling receptor complex”, “receptor complex”, and “extracellular space” in the category of CC, and “signaling receptor activator activity”, “receptor ligand activity”, “receptor regulator activity”, “G protein-coupled receptor binding”, and “chemokine activity” in the category of MF.

**Figure 4:**
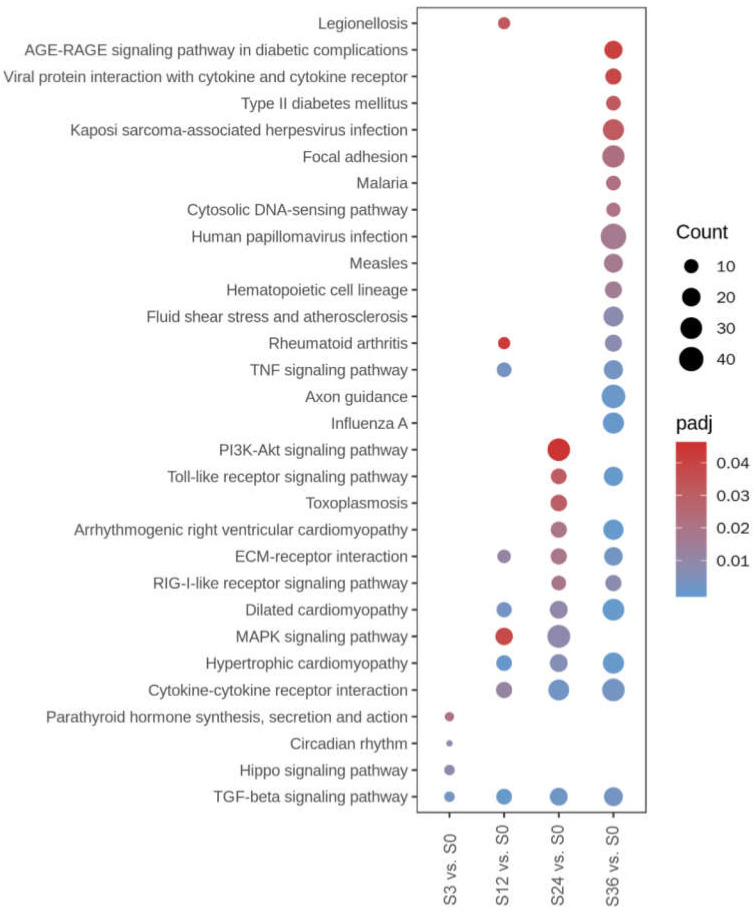
Significantly enriched KEGG pathways revealed by the KEGG pathway’s enrichment analysis of DEGs. The size of the dots indicates the number of DEGs enriched in the term (Count). The color of the dots indicates the adjusted *p*-value (padj), with a bluer color indicating a smaller padj value. Number of the enriched pathways increased with time, with the “TGF-beta signaling pathway” enriched from 3 to 36 hpi and “Cytokine-cytokine receptor interaction” enriched from 12 to 36 hpi.

For KEGG pathway enrichment (Figure 4), only four significantly enriched KEGG pathways are found at group S3 vs. S0, including “TGF-beta signaling pathway” and “Hippo signaling pathway”. The number of the significantly enriched KEGG pathways in group S12 vs. S0 increased to nine, including “TGF-beta signaling pathway”, “TNF signaling pathway”, and “Cytokine-cytokine receptor interaction”. For group S24 vs. S0, 11 significantly enriched KEGG pathways were identified, including “TGF-beta signaling pathway”, “Cytokine-cytokine receptor interaction”, “RIG-I-like receptor signaling pathway”, and “Toll-like receptor signaling pathway”. The number of significantly enriched KEGG pathways at group S36 vs. S0 increased significantly to 23. Most of the significantly enriched KEGG pathways at group S24 vs. S0 still enriched at group S36 vs. S0, including the four enriched KEGG pathways described above. The other exclusively significantly enriched KEGG pathways that related to virus infection at group S36 vs. S0 included “Influenza A”, “Human papillomavirus infection”, “Kaposi sarcoma-associated herpesvirus infection”, and “Viral protein interaction with cytokine and cytokine receptor”.

The KEGG pathway “Cytokine-cytokine receptor interaction” was significantly enriched in the groups of S12 vs. S0, S24 vs. S0, and S36 vs. S0. The DEGs in the pathway were further collected in Table S6. Expression of 14 cytokine and cytokine receptor-related genes were down-regulated at the examined time points. For example, the expression of *bmp6* (encoding bone morphogenetic protein 6) and *tgfb3* (encoding transforming growth factor beta-3 proprotein) were significantly down-regulated from 12 hpi to 36 hpi. Expression of *il11a* (encoding interleukin 11a) were significantly down-regulated at the four examined time points. Another 19 cytokine and cytokine-receptor-related genes were up-regulated, especially at 36 hpi. The top up-regulated cytokine genes included *LOC122880389* (encoding C-X-C motif chemokine 11-like, CXCL11), *LOC122888056* (encoding C-X-C motif chemokine 6-like, CXCL6), *LOC122875308* (encoding C-C motif chemokine 4, CCL4), *LOC122875929* (encoding C-X-C motif chemokine 10, CXCL10), *LOC122884604* (encoding C-X-C motif chemokine 9, CXCL9), and *LOC122873767* (encoding interleukin-8, IL8), while the top up-regulated cytokine receptor genes included *LOC122881975* (encoding C-X-C chemokine receptor type 3-like, CXCR3), *LOC122881976* (encoding C-X-C chemokine receptor type 3-like, CXCR3), and *LOC122885641* (encoding C-X-C chemokine receptor type 2, CXCR2).

Expression of several cytokine and cytokine-receptor-related genes, encoding for CXCL11, CXCR2, CXCL6, IL11a, IL8, CXCL10, CXCL9, IL12ba, and IL15, were further examined by RT-qPCR in SCRV-infected cells (Figure S1). The genes encoding for CXCL6, IL8, CXCL9, CXCL11, and CXCR2 were all up-regulatory as expressed from 3 hpi, and CXCL10 was up-regulated from 12 hpi. Up-regulation of *IL12ba* was not obvious until 36 hpi. The other two examined genes, IL11a and IL15, were down-regulatory expressed after SCRV infection. Although the elevated levels of several genes, such as CXCL11, were higher than those detected in transcriptome analysis, the changing trend of the genes between RT-qPCR and transcriptome is consistent. Different expression levels detected between RT-qPCR and transcriptome could be caused by sample collection.

Considering the important roles of IFN in antiviral responses, the significantly enriched DEGs on PRRs (Pattern Recognition Receptors), IFN, and IFN stimulated genes (ISGs) were collected in Table S7 and analyzed. These genes include PRRs (*dhx58*, encoding LGP2; *ifih1*, encoding MDA5; *nlrc5*; *ptx3a*), interferon-regulatory factors (IRFs) (*irf1b*, *irf3*, *irf7*, *irf9*, and *irf10*), and ISGs. In the transcriptome analysis, these genes were not significantly up-regulated at 3 hpi, and most of these were significantly up-regulated at 24 and 36 hpi.

Expression of the IRFs and some ISGs was further examined by RT-qPCR in SCRV infected cells. As shown in Figure S2, for the IRFs, the genes encoding for IRF1b and IRF3 were up-regulated from 3 hpi, *irf9* and *irf10* were up-regulated from 12 hpi, and *irf7* was up-regulated from 24 hpi. For the ISGs, most of them were significantly up-regulated from 12 hpi, while the genes encoding for viperin and IFIT1 were up-regulated from 3 hpi. The two ISGs also had the highest elevation during SCRV infection.

### 3.4. Overexpression of ISGs Inhibited SCRV Replication in Fish Cells

**Figure 5:**
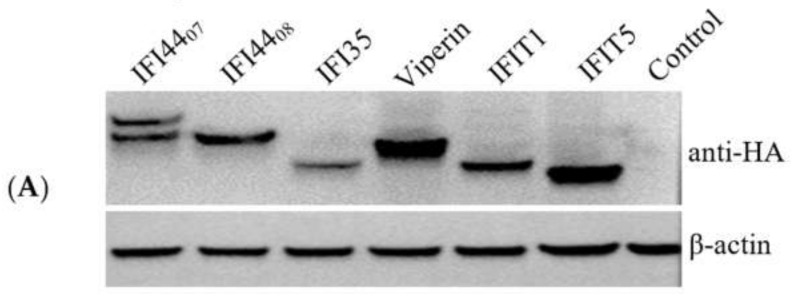

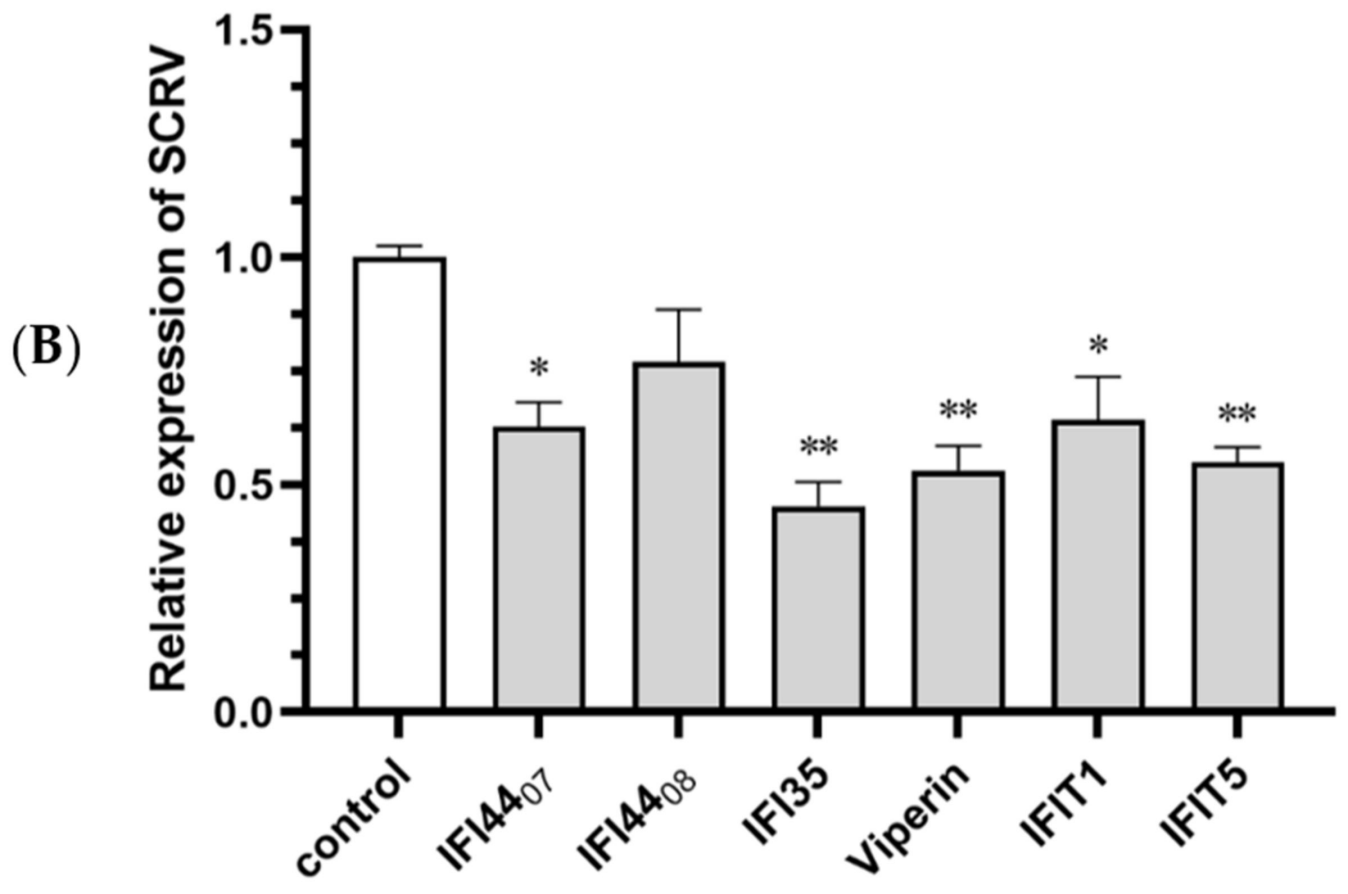
Antiviral effect of heterologously expressed ISGs on SCRV infection. (**A**) Western blot analysis of the expression of ISGs in FHM cells. The IFI44_07_, IFI44_08_, IFI35, Viperin, IFIT1, and IFIT5 of SCSC cells were successfully expressed in FHM cells. (**B**) RT-qPCR analysis of the expression of the SCRV gene in the ISGs expressed FHM cells. The expression level of the control was set as 1. * *p* < 0.05, ** *p* < 0.01.

To investigate the effect of these significantly up-regulated ISGs on SCRV infection, the ORFs encoding six ISGs (IFI44_07_, IFI44_08_, IFI35, Viperin, IFIT1, and IFIT5) were cloned successfully from SCRV-infected SCSC cells. A 2 × HA tag was fused on the N-terminal of these ISGs in favor of the expression detection. FHM cells were used in the overexpression assay for its high transfection efficiency. As shown in Figure 5A, the ISGs were expressed successfully in transfected FHM cells, although there were two bands for IFI44_07_. SCRV replication was detected using RT-qPCR in the overexpressed cells. As shown in Figure 5B, the expression of the SCRV gene was significantly inhibited in IFI44_07_-, IFI35-, Viperin-, IFIT1-, and IFIT5-overexpressed FHM cells. Among them, IFI35 had the highest inhibition efficiency (more than 50%), although Western blotting showed that the expression level of IFI35 was the lowest. In general, the overexpression of several ISGs coming sfrom SCSC inhibited SCRV replication in FHM cells.

## 4. Discussion

Recently, we established the SCSC cell line from the *S. chuatsi* skin tissue, which showed high sensitivity to several aquatic animal viruses [13]. In the present study, to explore the changes in gene expression profiles of SCSC cells after SCRV infection, a transcriptome analysis was performed. The results showed that few transcriptomic responses were induced at 3 hpi. A relatively strong response started at 12 hpi. The small number of the DEGs at 3 hpi affected the Go and KEGG pathway enrichment. Thus, we focused on the DEGs and enriched the Go and KEGG pathways at 12, 24, and 36 hpi.

The Go terms related to the stimulus appeared at 12 hpi, including cellular response to chemical stimulus/organic substance/endogenous stimulus, which also appeared at 24 and 36 hpi, indicating that the cells were responding to viruses invading. From the enriched KEGG pathways, the pathways that were enriched at 12, 24, and 36 hpi included ECM-receptor interaction, dilated cardiomyopathy, hypertrophic cardiomyopathy, cytokine–cytokine receptor interaction, and TGF-beta signaling pathway. The pathways that related to innate immune response appeared at 24 hpi, such as Toll-like receptor signaling pathway.

The fact that the significantly enriched KEGG pathways at group S36 vs. S0 included “Influenza A”, “Human papillomavirus infection”, “Kaposi sarcoma-associated herpesvirus infection”, and “Viral protein interaction with cytokine and cytokine receptor” could be due to the KEGG database that was searched in the present study. The pathway names in the KEGG database are usually assigned with the terms used in mammalian research. For example, the pathway “Influenza A” included several molecules or signaling pathways that were involved in influenza A virus infection, including many immune signaling pathways. The DEGs related to the immune response in the present study are probably homologs of the genes in the pathway “Influenza A”, which made the KEGG pathway enriched in the study. Checking the DEGs enriched in the KEGG pathway “Influenza A” in the present study verified this speculation. The DEGs are not genes of the human viruses but host genes related to virus infection. Such KEGG pathways, including “Herpes simplex infection”, were also found in transcriptome analysis of fish virus infection, such as grass carp reovirus and viral hemorrhagic septicemia virus infection [17,18].

In terms of the enriched pathways and the regulated immune-related genes, one feature was the up-regulation of cytokine and cytokine receptor-related genes. Among them, there were several groups of cytokine-cytokine receptors, for example, the CXCL9/10/11 and CXCR3. As a chemokine receptor, CXCR3 has three ligands CXCL9, CXCL10, and CXCL11, in which the CXCL11 has the strongest affinity [19]. CXCL11-CXCR3 has functions in chemotaxis and the regulation of T cells. The other group is CXCL6/IL8 and its receptors. IL8, also called CXCL8, is a member of the CXC chemokine family. As a proinflammatory chemokine, IL8 has chemotactic activity on neutrophils and T lymphocytes [20]. IL8 has two cell surface receptors, CXCR1 and CXCR2, in human cells [21]. The CXCR2 was also significantly up-regulated in SCRV-3h, which indicated that the IL8-CXCR2 signal transduction played important roles in SCRV infection.

Innate immune responses rely on the recognition of pathogen-associated molecular patterns (PAMPs) by the PRRs [22]. It has been reported that MDA5 (melanoma differentiation-associated gene 5) and LGP2 (DExH-box helicase 58) belong to the RIG-I-like receptors and can recognize and bind RNA [23]. In the present study, the RIG-I-like receptor (RLR) signaling pathway was significantly enriched from 24 hpi, including the up-regulation of the genes encoding for MDA5 and LGP2. It revealed that MDA5 and LGP2 participated in the response to SCRV infection in SCSC cells, which was in accordance with the report that members of RLRs were involved in fish against rhabdovirus infection [24,25]. Recognition of RNA by RLRs can induce downstream IFN responses and the expression of related antiviral genes [25]. There was also other PRRs that significantly up-regulated, such as *nlrc5* encoding NLRC5 (NLR Family CARD Domain Containing 5). NLRC5 has been reported to be a negative regulator in NF-κB signaling pathway and activator of MHC class I genes [26].

IFNs are a family of cytokines that play critical roles against virus invading in innate immunity, whose expression is regulated by IRFs [27,28]. The IFN response resists virus infection by inducing numerous ISGs [29]. In the present study, the up-regulation of five IRFs (IRF1/3/7/9/10) was observed in SCRV-infected SCSC cells. It has been reported that fish contain 11 IRFs [30]. IRF3 and IRF7 are the two main regulators of IFN expression, which are triggered by PRRs [31]. IRF1 has been identified to activate type I IFNs and was induced by many RNA viruses to contribute to the activation of innate immune responses [32]. IRF9 is a component of ISGF3 (Interferon-stimulated gene factor 3) that is essential for IFN signaling and ISGs expression [33]. IRF10 is found only in birds and fish, which has been identified as a negative regulator of the IFN responses [34]. The upregulation of IRF10 in SCSC cells indicates a balance of the cells’ antiviral responses.

ISGs are genes induced during IFN response, which have functions in antiviral defense, antiproliferative activities, and stimulation of adaptive immunity [35]. In the present study, several ISGs were up-regulated in SCRV-infected SCSC cells. We then cloned six ISGs (IFI44_07_, IFI44_08_, IFI35, Viperin, IFIT1, and IFIT5) from SCSC cells and examined the antiviral effect in overexpressed FHM cells. The function of the IFI44 in mammalian cells is divergent. It has been reported that IFI44 restricted the replication of respiratory syncytial virus but supported influenza A virus replication [36,37]. The upregulation of the IFI44-like gene has been reported in *Carassius auratus* herpesvirus-infected gibel carp [38]. The present transcriptomic analysis revealed the upregulation of different transcripts of IFI44-like genes in SCRV-infected SCSC cells. One of the IFI44-like genes (IFI44_07_) showed antiviral capacity in FHM cells in our overexpression assay. It should be noted that there were two protein bands in IFI44_07_ over-expressed cells. Because the 2 × HA tag was fused on the N-terminal of the ISG, the two protein bands could not be fround from a different translation start site. We speculated that the two bands might be caused by the processing of mRNA or translated protein, but it needs to be proven in future studies, as do the functions of the two products.

IFI35 (also known as IFP35) has been reported as a proinflammatory factor during cellular infection [39]. For fish IFI35, grouper IFI35 has been reported to inhibit fish nodavirus replication [40], and rock bream IFI35 has been reported to inhibit the transcription of viral hemorrhagic septicemia virus [41]. However, it has been reported that the overexpression of IFI35 in striped snakehead cells promoted the replication of snakehead vesiculovirus [42]. It seems that fish IFI35 has different roles under different virus infections in different cells. Our study showed that the overexpression of *S. chuatsi* IFI35 inhibited the replication of SCRV in SCSC cells.

It has been reported that viperin has the ability to act against various viruses via multiple mechanisms [43]. The antiviral capacity of viperin has been reported in many fish species [44,45,46], and our study verified the antiviral ability of viperin from *S. chuatsi*. The IFN-induced protein with the tetratricopeptide repeats (IFITs) family contains several members including IFIT1, IFIT2, IFIT3, IFIT4, and IFIT5 [47]. IFIT1 (also known as ISG56) has been identified in crucian carp, tongue sole, olive flounder, and grouper for its antiviral functions [48,49,50,51]. There are few functional investigations of IFIT5 in fish compared to IFIT1. It has been reported that IFIT5 participates in antiviral responses in rainbow trout red blood cells [52].

In summary, the present study analyzes the important cellular responses and antiviral genes of a new cell line SCSC under SCRV infection by transcriptome and RT-qPCR, which revealed several key pathways in SCRV infection, such as the pathways of “Cytokine-cytokine receptor interaction” and “interferon related pathways”. Further functional analysis verified the major ISGs including IFI44_07_, IFI35, Viperin, IFIT1, and IFIT5 in anti-SCRV. Thus, these results provide new information in the research on SCRV infection and can be beneficial to the antiviral exploration in the aquaculture of Mandarin fish.

## Figures and Tables

**Table 1:** Summary of the SCSC transcriptome data.

Sample	Clean Bases (G)	Clean Reads	Q30 (%)	Mapped Reads	Number of Expressed Genes (Total: 26,250)
S0-1	10.38	34,722,124	95.30	31,642,860 (91.13%)	19,750
S0-2	10.06	33,621,806	96.32	31,263,781 (92.99%)	19,433
S0-3	10.26	34,323,454	95.08	31,612,089 (92.10%)	19,698
S3-1	9.21	30,812,244	95.72	28,566,266 (92.71%)	19,635
S3-2	9.43	31,548,020	95.38	29,075,968 (92.16%)	19,427
S3-3	8.06	26,984,172	94.74	25,086,243 (92.97%)	19,310
S12-1	10.93	36,557,790	95.58	34,077,971 (93.22%)	19,387
S12-2	9.89	33,122,514	94.68	30,936,755 (93.40%)	19,598
S12-3	12.28	41,083,674	95.54	34,742,929 (84.57%)	19,968
S24-1	10.63	35,573,294	95.39	33,085,377 (93.01%)	20,382
S24-2	10.78	36,094,052	94.61	33,393,082 (92.52%)	20,269
S24-3	10.28	34,395,705	95.17	31,946,646 (92.88%)	20,274
S36-1	10.64	35,594,041	95.45	33,164,474 (93.17%)	20,465
S36-2	8.93	29,865,397	94.00	27,871,748 (93.32%)	20,325
S36-3	9.47	31,670,223	95.60	29,681,283 (93.72%)	20,291

## Data Availability

The sequencing data were deposited in the Science Data Bank (https://www.scidb.cn/en, accessed on 10 October 2022) under the accession number: 31253.11.sciencedb.03063 or can be viewed via the link https://www.scidb.cn/en/s/qMjMbu, accessed on 10 October 2022.

## Supplementary Materials

The following supporting information can be downloaded at https://www.mdpi.com/article/10.3390/microorganisms10122464/s1: Figure S1: Expression of the representative DEGs of “chemokine and chemokine receptors” in SCRV-infected SCSC cells revealed by RT-qPCR; Figure S2: Expression of representative DEGs related to the IFN-signaling pathway in SCRV-infected SCSC cells as revealed by RT-qPCR; Table S1: Genes and primers used in the study; Table S2: S0_vs_S3.GO.enrichment; Table S3: S0_vs_S12.GO.enrichment; Table S4: S0_vs_S24.GO.enrichment; Table S5: S0_vs_S36.GO.enrichment; Table S6: Enriched DEGs in cytokine and cytokine receptor pathway; Table S7: Enriched DEGs related to PRR, IRF, and ISG.
