# Supplementary material for: Siniperca chuatsi Rhabdovirus (SCRV)-Induced Key Pathways and Major Antiviral Genes in Fish Cells
Source: Microorganisms. 2022 Dec 13;10(12):2464. doi: 10.3390/microorganisms10122464 (PMC9788611; doi:10.3390/microorganisms10122464)
Supplement: Supplementary file 1 [file microorganisms-10-02464-s001.zip › Figure S1.pdf]

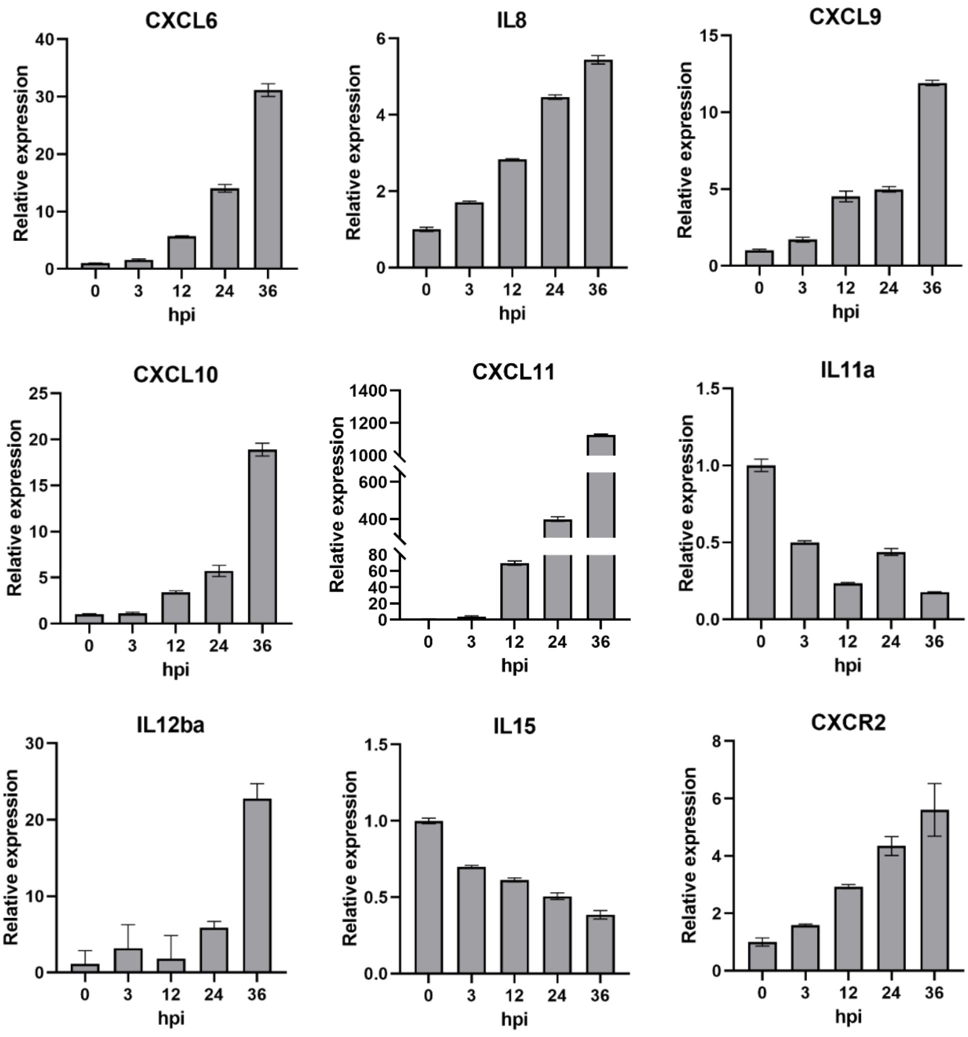

Figure S1. Expression of the representative DEGs of "chemokine and chemokine receptors" in SCRV infected SCSC cells revealed by RT-qPCR. The encoded proteins by the DEGs were shown in the figure. Expression level at 0 hpi was set as 1 in each figure.
