# Supplementary material for: Siniperca chuatsi Rhabdovirus (SCRV)-Induced Key Pathways and Major Antiviral Genes in Fish Cells
Source: Microorganisms. 2022 Dec 13;10(12):2464. doi: 10.3390/microorganisms10122464 (PMC9788611; doi:10.3390/microorganisms10122464)
Supplement: Supplementary file 1 [file microorganisms-10-02464-s001.zip › Table S1 genes and primers used in RT-qPCR.pdf]

Table S1 The genes and primers used in RT-qPCR, gene cloning, and overexpression assays in the study.

Gene	Primers (5'-3')	application
CXCL6	TGTCACCCTAAAGAGGGGCT	RT-qPCR
	GCTCAGTTGGTCTGGCTGTA	
IL8	GAGAGCAAACCCATAGGCCG	
	TGGGTCCAGGCAAACCTCTT	
CXCL9	GAGCTCACGGTCTACCCAAA	
	TCACGACCCAACCTATGAGC	
CXCL10	TTTCTCACCTGCCTGCTTGT	
	TGACTGGCTCTGCCTTGATG	
CXCL11	TGTGTAATCGTGTCAGGAGCAA	
	CCAGGCAATAACGAAGGCTG	
IL11a	CATCTCCAGTACCACAGCGG	
	CAAATGCATGCTCCCTCAGC	
IL12ba	ACTGTATTTCCCGGGATAGCG	
	AGTCCAGTGGCTCGTAGGAT	
IL15	GCCCCTGACTTGAAGTGGTT	
	CCTTGAACGGTCTCCCACTC	
CXCR2	ACACCGCACCACTTTGTAGT	
	ACTGTGAAGCAGGCCCATAC	
IRF1b	GGGGGTTCCTGAGCAATGAA	
	AAGCTGTTCCAGAGGTCGTC	
IRF3	AGAATCCACTGCCAACCAGG	
	GCAACGCGTGTCTACCAATC	
IRF7	CCCATGGAAGCACAACTCCA	
	TTTCCACCTGGCCTTGTCAG	
IRF9	ATCTCCGAGCCCTACAAGGT	
	CATCGTCGCTCTCCGATTCA	
IRF10	TGTCCCCTTGGGAAACGAAC	
	ACACCCCTCTCCAGATGACA	
Spock2	CTGCGATGAGGACGGCTATT	
	CCACGAATCCTAGATCCGGC	
IFI44 LOC122878107	TCCATAGGTGATCTCAGCATAGC	
	AGGAGAATCCGAGCCTGAGT	
IFI44 LOC122878108	CCAGCATGGCTACAAACGTC	
	GAATCCGAGCCTGAGTCACC	
Viperin	AGCCTGCAAGGGAACAACTT	
	AAGTTTGAGGCCCCTCTTGG	
IFI35	CAGCGCTCCTACAAAGACCA	
	CTGTCGACCCCATGAACACA	
IFIT1	ACCAGCCAAGTCATAGGGTT	
	AGTGAACGGCGAGGTACAAG	
IFIT5	CAGCACTCGACTACAGGACC	
	TTGAGAAGTGACACCGCCTC	
SCSC-β-actin	GAGAGGGAAATCGTGCGTGA	
	CATACCGAGGAAGGAAGGCTG	
FHM-β-actin	GAATCCCAAAGCCAACAG	
	GGAAGAGCATAACCCTCATAG	
IFI44	ATGGATGAACGAAATAAATACAGCTTCAATGG	Gene cloning

LOC122878107	TTAGTCTATCTTTGTCCCTGTGTATTGTGG	
IFI44	ATGCAGAATCAAACTGGATCTGGATTCTCG	
LOC122878108	TTAGTCTATCTTTGTCCCTGTGTATTGTGG	
Viperin	ATGCAGTTCTCCCCTGTGGTC	
	TCACCACTGCAGCTTCATGTTGGCT	
IFI35	ATGTCTTCAGATGAGGATTTCTCTATAGTG	
	CTACTCTCCGTTTGGTGAGACGC	
IFIT1	ATGATGAGTGCTGCTCAGAGTCCGAC	
	CTATGGCTCTTGCAGGTTGGCCAGA	
IFIT5	ATGAGCAGCACTCTTCACTCCAGACTG	
	TCATCCCTGCAGCTCCATGCGCAGCTC	
IFI44	CGCTGAATTCTGCAGATATATGGATGAACGAAATAAATACAG	Overexpression
LOC122878107	CGCCACTGTGCTGGATATTTAGTCTATCTTTGTCCCTG	
IFI44	CGCTGAATTCTGCAGATATATGCAGAATCAAACTGGATC	
LOC122878108	CGCCACTGTGCTGGATATTTAGTCTATCTTTGTCCCTG	
Viperin	CGCTGAATTCTGCAGATATATGCAGTTCTCCCCTGTG	
	CGCCACTGTGCTGGATATTCACCACTGCAGCTTCATG	
IFI35	CGCTGAATTCTGCAGATATATGTCTTCAGATGAGGATTTC	
	CGCCACTGTGCTGGATATCTACTCTCCGTTTGGTGAG	
IFIT1	CGCTGAATTCTGCAGATATATGATGAGTGCTGCTCAG	
	CGCCACTGTGCTGGATATCTATGGCTCTTGCAGGTTG	
IFIT5	CGCTGAATTCTGCAGATATATGAGCAGCACTCTTCACTC	
	CGCCACTGTGCTGGATATTCATCCCTGCAGCTCCAT	
SCRV-RT-F	GGGCTGGATGATAGACGATTG	RT-qPCR
SCRV-RT-R	TGGCGGAGGTGCTTGATATGG	
