# Supplementary material for: Siniperca chuatsi Rhabdovirus (SCRV)-Induced Key Pathways and Major Antiviral Genes in Fish Cells
Source: Microorganisms. 2022 Dec 13;10(12):2464. doi: 10.3390/microorganisms10122464 (PMC9788611; doi:10.3390/microorganisms10122464)
Supplement: Supplementary file 1 [file microorganisms-10-02464-s001.zip › Table S6. Enriched DEGs in cytokine and cytokine receptor pathway.pdf]

Table S6. Enriched DEGs in Cytokine-cytokine receptor interaction pathway

gene	Possible function/pathway	Expression change fold (log_2FC)			
		3 h	12 h	24 h	36 h
bmp16	TGF_BETA_2 domain-containing protein			-1.16	-1.38
bmp6	Bone morphogenetic protein 6		-1.28	-1.27	-1.29
bmpr1ba	Bone morphogenetic protein receptor type-1Ba			-1.39	-2.01
tgfb3	Transforming growth factor beta-3 proprotein		-1.39	-1.71	-2.07
ccl44	C-C motif			-1.88	-2.66
cxcl12b	C-X-C motif chemokine 12			-1.35	-2.10
gdf10b	Growth/differentiation factor 10			-1.03	-1.55
il11a	Interleukin 11a	-1.21	-1.34	-1.85	-1.88
il15l	Interleukin-15		-1.35	-1.93	-2.35
LOC122872036	Inhibin beta B chain		-1.72	-2.01	-2.86
LOC122872371	Activin receptor type-2A			-1.11	-1.50
LOC122874983	Intercrine alpha family (small cytokine C-X-C)				-1.78
mpl	Thrombopoietin receptor		-1.44	-1.57	-1.47
osmr	Oncostatin-M-specific receptor subunit beta				-1.58
LOC122880389	C-X-C motif chemokine 11-like				7.76
LOC122888056	C-X-C motif chemokine 6-like				6.91
LOC122875308	C-C motif chemokine 4				4.51
LOC122875929	C-X-C motif chemokine 10				3.28
LOC122884604	C-X-C motif chemokine 9				3.21
LOC122873767	Interleukin-8				3.16
LOC122881975	C-X-C chemokine receptor type 3-like				2.84
LOC122876711	Interleukin-1 beta				2.44
LOC122881976	C-X-C chemokine receptor type 3-like				1.88
LOC122883012	Tumor necrosis factor receptor superfamily member 5				1.72
LOC122885641	C-X-C chemokine receptor type 2				1.71
il12rb2l	Interleukin 12 receptor, beta 2a, like<br>Interleukin-6 receptor subunit beta				1.53
il15ra	Interleukin-15 receptor subunit alpha				1.49
il12ba	Interleukin-12 subunit beta				1.45
bmpr1bb	Bone morphogenetic protein receptor type-1Bb				1.44
si:ch211-112c15.8	Tumor necrosis factor receptor superfamily member 1A				1.21
lepr	Leptin receptor				1.16
LOC122883385	Interleukin-17 receptor E				1.16
csf1ra	Macrophage colony-stimulating factor 1 receptor 1				1.14
