# Supplementary material for: Siniperca chuatsi Rhabdovirus (SCRV)-Induced Key Pathways and Major Antiviral Genes in Fish Cells
Source: Microorganisms. 2022 Dec 13;10(12):2464. doi: 10.3390/microorganisms10122464 (PMC9788611; doi:10.3390/microorganisms10122464)
Supplement: Supplementary file 1 [file microorganisms-10-02464-s001.zip › Table S7. Enriched DEGs related to PRR, IRF, and ISG.pdf]

Table S7. Enriched DEGs related to PRR, IRF, and ISG

| | gene | protein | Expression change fold (log2FC) | | | |
|---|---|---|---|---|---|---|
| | | | 3 h | 12 h | 24 h | 36 h |
| PRR | dhx58 | Probable ATP-dependent RNA helicase DHX58 (LGP2) | | | 2.37 | 4.42 |
| | ifih1 | Interferon-induced helicase C domain-containing protein 1 (MDA5) | | | 1.06 | 2.05 |
| | nlrc5 | NOD-like receptor C5 | | | 3.28 | 4.61 |
| | ptx3a | Pentraxin-related protein PTX3 | | | 2.32 | 2.72 |
| IRF | irf1b | Interferon regulatory factor 1 | | 1.60 | 2.29 | 2.46 |
| | irf3 | Interferon regulatory factor 3 | | 1.22 | 2.03 | 2.89 |
| | irf7 | Interferon regulatory factor 7 | | | | 1.57 |
| | irf9 | Interferon regulatory factor 9 | | | | 1.11 |
| | Irf10 | Interferon regulatory factor 10 | | | | 2.01 |
| ISG | LOC122871629 | E3 ubiquitin/ISG15 ligase TRIM25 | | | 4.64 | |
| | spock2 | Testican-2 (IFN induced) | | | 4.02 | |
| | LOC122868453 | Interferon-induced protein 44 | | | 3.7 | |
| | LOC122878108 | Interferon-induced protein 44 | | | 3.00 | 5.26 |
| | LOC122878107 | Interferon-induced protein 44 | | | | 4.76 |
| | rsad2 | Radical S-adenosyl methionine domain-containing protein 2 (IFN induced) (Viperin) | | | 3.32 | 4.07 |
| | LOC122862285 | Interferon-induced GTP-binding protein Mx | | | 3.27 | |
| | LOC122878212 | interferon-induced protein with tetratricopeptide repeats 1-like | | | 2.48 | 5.19 |
| | LOC122868174 | Interferon-induced protein with tetratricopeptide repeats 5-like | | | | 1.35 |
| | ifi35 | Interferon-induced 35 kDa protein homolog | | | | 2.30 |
| | LOC122869735 | Erythroid membrane-associated protein E3 ubiquitin-protein ligase TRIM21 | | | 4.27 | 6.87 |
